# Differences Between Patients With Multiple System Atrophy With Predominant Parkinsonism and Parkinson's Disease Based on fNIRS and Gait Analysis

**DOI:** 10.1111/cns.70342

**Published:** 2025-03-26

**Authors:** Mengxi Gao, Heng Zhang, Aidi Shan, Yongsheng Yuan, Xingyue Cao, Lina Wang, Caiting Gan, Huimin Sun, Shiyi Ye, Chenghui Wan, Youyong Kong, Kezhong Zhang

**Affiliations:** ^1^ Department of Neurology The First Affiliated Hospital of Nanjing Medical University Nanjing China; ^2^ Jiangsu Provincial Joint International Research Laboratory of Medical Information Processing School of Computer Science and Engineering, Southeast University Nanjing China

**Keywords:** dorsolateral prefrontal cortex, functional near‐infrared spectroscopy, gait impairments, multiple system atrophy with predominant parkinsonism, Parkinson's disease

## Abstract

**Objective:**

To investigate the differences in gait parameters and cortical activity during a single‐task walking (STW) and cognitive dual‐task walking (DTW) between multiple system atrophy with predominant parkinsonism (MSA‐P) and Parkinson's disease (PD).

**Methods:**

24 MSA‐P patients, 20 PD patients, and 22 healthy controls (HCs) were enrolled. Gait parameters were collected using a portable inertial measurement unit system, and the relative change of oxyhemoglobin (ΔHbO_2_) in the bilateral frontal and sensorimotor cortex was obtained by functional near‐infrared spectroscopy during walking with and without cognitive tasks.

**Results:**

MSA‐P patients had increased step length variability and higher ΔHbO_2_ in the right dorsolateral prefrontal cortex (DLPFC), relative to PD patients and HCs during the DTW condition. Meanwhile, MSA‐P patients exhibited higher step length variability and ΔHbO_2_ in the right DLPFC during DTW compared to STW. Furthermore, mild negative correlations were found between the ΔHbO_2_ in the right DLPFC and step length, while there was a mild positive correlation between ΔHbO_2_ and step length variability during the DTW condition. Notably, receiver operating characteristic (ROC) curve analysis uncovered that the areas under the curve (AUCs) of the ΔHbO_2_ of the right DLPFC and step length variability during DTW were 0.798 (95% confidence interval [CI]: 0.651–0.945, sensitivity = 0.650, specificity = 0.958) and 0.721 (95% CI: 0.570–0.871, sensitivity = 0.625, specificity = 0.800), respectively.

**Conclusion:**

MSA‐P patients demonstrate more severe gait disturbance and increased DLPFC activity compared with PD patients and HCs. Gait parameters and cortical activity could be a potential features discerning MSA‐P patients and PD patients.

## Graphical abstract

We found a significant interaction effect between group and condition in the right dorsolateral prefrontal cortex. Bonferroni post hoc tests indicated MSA‐P patients exhibited a significant increase in ΔHbO_2_ compared to PD patients during the DTW condition.

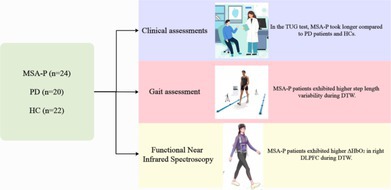

## 1 Introduction

Multiple system atrophy (MSA) is a rapidly progressing neurodegenerative disease. It can be categorized into two variants based on clinical symptoms: the Parkinsonian variant (MSA‐P) and the cerebellar variant (MSA‐C) [1]. Initially, patients with MSA‐P often exhibit clinical symptoms similar to those of Parkinson's disease (PD), making it challenging to distinguish between MSA‐P and PD. Moreover, MSA has an aggressive progression and a poorer prognosis, with a mean survival of 8–9 years [2]. Therefore, making an early and reliable distinction between PD and MSA‐P is crucial to develop individualized treatment plans promptly. Doing so can enhance both the survival rate and quality of life for patients, as well as alleviate the psychosocial and economic burdens faced by patients and their families [3].

There is a dearth of differential diagnostic biomarkers for distinguishing MSA and PD, particularly during the early stages. Currently, imaging techniques serve as the primary diagnostic method, despite suboptimal sensitivity [4]. Consequently, the diagnosis may be substantially delayed, sometimes by several years [5]. Ongoing research is actively exploring other potential diagnostic biomarkers, such as gait parameters, which reflect the progression of movement disorders [6]. Recent studies suggested that MSA‐P patients showcased greater gait variability, larger total anterior–posterior and lateral center of pressure (COP) movement, and a widened base of support in comparison to PD patients [7, 8]. In addition, previous research has shown that PD patients experience exacerbated gait impairments when engaging in concurrent cognitive tasks during walking, possibly because of abnormal cortical activity [9]. Nevertheless, the differences in cortical activity changes during walking have not been studied in patients with MSA‐P and PD. Hence, we hypothesize that detecting the changes in cortical activation during complex walking can reveal the differences in the neurophysiological mechanisms of gait disorders in patients with PD and MSA‐P.

Inertial measurement units (IMUs) have demonstrated high sensitivity and specificity as adjunct devices for evaluating gait performance. Several studies have already examined the feasibility and clinical applicability of IMUs in patients with PD and MSA [8, 10]. Functional near‐infrared spectroscopy (fNIRS) is a non‐invasive imaging device that records cortical activity by measuring changes in concentrations of oxygenated hemoglobin (HbO_2_) and deoxygenated hemoglobin (HHb) [11]. Compared to functional magnetic resonance imaging (fMRI), fNIRS offers higher temporal resolution, and it surpasses electroencephalogram (EEG) in terms of spatial resolution [12, 13]. Mobile fNIRS, a more portable and flexible variant, has also gained attention [14]. In recent years, several studies have utilized fNIRS imaging technology to examine cortical activity during walking in both healthy individuals and PD patients. These studies have revealed higher activation of the prefrontal and sensorimotor cortex in PD patients while performing cognitive tasks (e.g., serial subtraction) during walking, compared to walking without cognitive tasks, as well as compared to healthy individuals [15, 16, 17, 18]. The prefrontal cortex (PFC) is associated with cognitive regulation, working memory, and information synthesis, and all of these are necessary for sophisticated behavior [19]. Additionally, walking is influenced by posture control, which is primarily controlled by the sensorimotor cortex [20]. Based on this evidence, we believe fNIRS holds promise as a tool for differentiating MSA‐P from PD patients. Consequently, we investigated gait parameters and cortical activity in PD and MSA‐P patients during single‐task walking (STW) and cognitive dual‐task walking (DTW) to explore their potential for this differentiation. We hypothesized that MSA‐P patients would show increased cortical activity during complex task walking compared to PD patients because walking capabilities in MSA‐P may rely on much more compensation from related motor cortical regions.

## 2 Materials and Methods

### 2.1 Subjects

Between 2021 and 2023, 24 MSA‐P patients, 20 PD patients, and 22 healthy controls (HCs) were recruited from the outpatients of the Neurology Department at the First Affiliated Hospital of Nanjing Medical University. The investigation was approved by the hospital's Ethics Committee, and written informed consent was obtained from all participants (2022‐SR‐603). MSA‐P patients were diagnosed according to the second consensus diagnosis of MSA [1], while PD patients were diagnosed based on the Movement Disorder Society (MDS) Clinical Diagnostic Criteria for PD [21]. The inclusion criteria for all participants were as follows: (1) aged between 50 and 80 years; (2) right‐hand dominant; (3) able to walk independently without assistive devices; (4) on a stable medication regimen for at least 4 weeks; (5) in an “ON” medication state during the experiment. Participants were excluded if they met any of the following criteria: (1) significant cognitive impairment, as indicated by a Mini‐Mental State Examination (MMSE) score < 24 [22]; (2) inability to stand or walk continuously for 90 s; (3) presence of gait disorders, such as severe postural hypotension or freezing of gait (MDS‐Unified PD Rating Scale [MDS‐UPDRS] Section 2.14 score ≥ 1); (4) other factors affecting gait, such as musculoskeletal disorders, hip replacement, uncorrected vision, or vestibular problems; and (5) inability to follow instructions. Additionally, HCs were recruited, matching their age, sex, years of education, and general cognition with the PD and MSA‐P groups. The exclusion criteria for HCs were consistent with those mentioned above.

### 2.2 Clinical Assessments

All participants were evaluated in their on‐medication states due to the possibility of gait disorder during the close‐medication period and the potential difficulty in cooperation with the study. To account for variations in medication use, we recorded each individual's medication regimen and converted it into a levodopa equivalent daily dose (LEDD), ensuring no significant differences between the two groups. The study subjects' socio‐demographic characteristics, such as gender, age, education level, medical history, and disease duration, were collected. The Time Up and Go (TUG) test was utilized to assess the severity of gait impairments. Disease severity was evaluated using the Hoehn‐Yahr (H‐Y) staging and MDS‐Unified PD Rating Scale part III (MDS‐UPDRS III) scores [23, 24]. Cognitive performance was evaluated using the Frontal Assessment Battery (FAB) [25] and MMSE. Psychopathological screening was conducted using the 14‐item Hamilton Anxiety Rating Scale (HAMA) [26] and the 24‐item Hamilton Depression Rating Scale (HAMD) [27].

### 2.3 Gait Assessment

All participants in the study underwent gait measurements using a portable IMU system (GYENNO Science) for objective assessment. IMUs were attached to the subjects' chest, back, wrists, thighs, ankles, and insteps using Velcro straps in a specific order. The mobility data collected wirelessly were then stored and analyzed on a laptop controlling the experimental setup. Two conditions, STW and DTW, were randomly assigned. In the STW condition, participants were instructed to walk at a comfortable pace while performing a 5‐m timed TUG test. In the DTW condition, participants were asked to simultaneously walk and carry out a cognitive task, specifically subtracting 7 from a randomly generated 3‐digit number in a serial manner. The order of the tasks was randomized for each participant. Each gait parameter was averaged over the 3 trials. The mean values and variability (expressed as the coefficient of variation, CV) of various spatial parameters were recorded for each individual. These parameters included step length, stride velocity, stride length, double support time, gait cycle, average duration of turning, and average velocity of turning, which were extracted from the whole test. The CV was calculated as the standard deviation divided by the mean, multiplied by 100%.

### 2.4 Functional Near‐Infrared Spectroscopy

A mobile fNIRS system (HuiChuang, China) was utilized in this study. The system employed continuous wave diodes with wavelengths of 730 and 850 nm to measure the optical intensities of HbO_2_ and HHb. The sampling rate was set at 11 Hz. A total of 8 fNIRS probes, consisting of 14 sources and 14 detectors, were positioned on specific areas. This configuration resulted in 35 distinct fNIRS channels [28]. The distribution of these 35 channels can be observed in Figure S1, while their corresponding coordinates are provided in Table S1. According to the coordinate information, 35 channels were divided into regions of interest (ROI) in the subjects' cerebral cortex, namely dorsolateral prefrontal cortex (DLPFC), pre‐motor area (PMA), supplementary motor area (SMA), the primary motor cortex (M1), primary somatosensory cortex, and frontopolar area. These placements are consistent with those used in the study by Zhang et al. [18]. The instrument employed the principle of three‐dimensional positioning and was based on the international 10/20 system for electrode placement. To ensure accurate data acquisition, a flexible headgear was used to securely fix the signal sources and detectors in place. Moreover, the average distance between the signal sources and detectors was set to 30 mm to maximize contact with the skin. To minimize interference from ambient light, participants wore black hoods. The quality of the acquired signals was assessed through visual inspection.

Cortical activity was recorded during two different walking conditions: STW, which involved walking back and forth along a 5 m pathway at a comfortable pace, and DTW. In the DTW condition, participants were instructed to perform a sequential subtraction task, subtracting seven from a randomly generated three‐digit number provided prior to each trial, while walking. Participants were asked to walk with no explicit indication for prioritization. There was no practice provided before completing the dual‐task trial. All participants turned to their left at the end of the 5 m pathway. Each task was performed three times in a pseudo‐randomized order. During the DTW condition, cognitive performance was measured and defined as the number of correct operations. Each task consisted of an initial 30‐s period of quiet standing followed by 90s of walking. The order of each condition was randomized between participants. The subtraction task was initiated while the participants were in a stationary position and continued throughout the walking activity. It is worth noting that participants were required to stand still for at least 1 min before each experiment to ensure a stable blood pressure baseline.

The fNIRS data processing protocol in this study followed established recommendations [29]. For subsequent analyses, we specifically utilized the concentration of HbO_2_ since it is widely recognized as the primary indicator for changes in cortical activity associated with walking [30] and offers enhanced sensitivity [31]. To preprocess the fNIRS signals, we employed the NirSpark software package (HuiChuang, China). Initially, physiological artifacts such as respiration, heart activity, and low‐frequency signal drift were corrected using a filter with cut‐off frequencies ranging from 0.01 to 0.1 Hz. Subsequently, motion artifacts were mitigated using a combination of moving standard deviation and cubic spline interpolation techniques. Furthermore, the light intensity was transformed into optical density, and the modified Beer–Lambert law was applied to convert the optical density signal into concentrations of HbO_2_ and HHb. To ensure consistency, we excluded the 5‐s intervals immediately preceding and following each task event. We marked when participants started walking. Additionally, we used the HbO_2_ when standing as the baseline HbO_2_ and subtracted it from the experimental task to evaluate the relative change in HbO_2_ (ΔHbO_2_) during the walking task [32].

### 2.5 Statistical Analysis

Mean values with standard deviations were used to present all data. Statistical analyses were performed using IBM SPSS v27.0 software, and the Shapiro–Wilks test was utilized to assess normality. A significance level of 5% was set for all analyses. Baseline demographic and clinical characteristics were evaluated using the *χ* ^2^ test and Fisher's exact test for categorical variables. Continuous variables were analyzed using one‐way analysis of variance (ANOVA), Kruskal–Wallis test, two‐sample *t*‐test, and Mann–Whitney test. Two‐way ANOVA was employed to examine differences in gait parameters and ΔHbO_2_, with a group (MSA‐P vs. PD vs. HCs) and condition (STW vs. DTW) as factors. Since the comparison of ΔHbO_2_ was performed across 9 ROI, a false discovery rate (FDR) correction was applied to the *p*‐values. Significant interactions identified through ANOVA were further explored using the Bonferroni post hoc test, which accounted for multiple comparisons (*p* value/Number of comparisons). Effect sizes were estimated using the *η* ^2^squared (pη^2^) statistic. Moreover, Spearman correlation analysis was conducted to examine associations between gait parameters and ΔHbO_2_. ROC curve analysis was performed to assess the ability of gait parameters and cortical activity to differentiate MSA‐P from PD patients. Cut‐off values were determined, including specificity, sensitivity, and area under the curve (AUC). The optimal cut‐off point was selected based on the maximization of Youden's index.

## 3 Results

### 3.1 Demographic and Clinical Characteristics

A total of 20 patients with PD, 24 patients with MSA‐P, and 22 HCs were enrolled in this study. Table 1 presents a summary of the demographic and clinical characteristics of all participants. No significant differences were observed in age, sex, education level, disease duration, MMSE scores, FAB scores, HAMA scores, HAMD scores, or cognitive performance among the groups. Furthermore, there were no significant differences in MDS‐UPDRS III scores or H&Y stage between MSA‐P and PD patients. However, in the TUG test, it was found that MSA‐P patients took longer to complete the task compared to PD patients (*p* = 0.011) and HCs (*p* < 0.001), while no significant differences were observed between PD patients and healthy controls (Table 1).

**TABLE 1:** Demographics and clinical characteristics in the disease groups.

Characteristics	MSA‐P	PD	HC	*p*	Post hoc (Bonferroni corrected)
*n*	**24**	**20**	**22**		
Age (y)	**61.22 ± 4.67**	**63.00 ± 8.02**	**60.55 ± 7.00**	**0.205** ^**a**^	
Sex (M/F)	**12/6**	**16/6**	**13/7**	**0.677** ^**b**^	
Education (y)	**11.72 ± 3.61**	**13.18 ± 2.46**	**12.35 ± 3.07**	**0.401** ^**c**^	
Disease duration (y)	**3.61 ± 2.65**	**3.84 ± 2.33**	**NA**	**0.766** ^**d**^	
MMSE	**28.72 ± 0.90**	**28.59 ± 0.85**	**28.90 ± 0.85**	**0.597** ^**c**^	
FAB	**15.33 ± 2.57**	**15.73 ± 2.07**	**16.65 ± 1.63**	**0.172** ^**c**^	
HAMA	**3.28 ± 2.14**	**3.00 ± 1.77**	**2.25 ± 0.91**	**0.421** ^**c**^	
HAMD	**2.61 ± 1.65**	**2.55 ± 1.77**	**1.60 ± 0.94**	**0.665** ^**c**^	
UPDRS III (ON state)	**16.45 ± 8.38**	**12.33 ± 2.51**	**NA**	**0.887** ^**e**^	
H&Y stage (ON state)	**(1–1.5) 4** **(2–2.5) 16** **(3–3.5) 4**	**(1–1.5) 14** **(2–2.5) 6** **(3–3.5) 0**	**NA**	**0.623** ^**c**^	
TUG (s)	**18.60 ± 4.81**	**14.46 ± 3.00**	**12.39 ± 2.39**	**< 0.001** ^**a**^,*	**MSA‐P>PD (*p* = 0.011)** **MSA‐P>HC (*p* < 0.001)**
Cognitive performance	**9.42 ± 3.36**	**10.20 ± 3.07**	**10.55 ± 3.38**	**0.502** ^**a**^	
LEDD, mg/day	**612.50 ± 78.71**	**570.51 ± 233.41**	**NA**	**0.386** ^**e**^	

*Note:* Values are presented as the mean ± standard deviation. *p* < 0.05 was considered statistically significant. Values in bold are marked as significant.

Abbreviations: F, female; FAB, frontal assessment battery; H & Y stage, Hoehn and Yahr clinical rating scale; HAMA, Hamilton Anxiety Scale; HAMD‐24, Hamilton Depression Scale‐24; HC, healthy control; LEDD, levodopa equivalent daily dose; M, male; MMSE, Mini‐Mental State Examination; MSA‐P, multiple system atrophy with predominant parkinsonism; NA, not applicable; PD, Parkinson's disease; s, second; TUG, Time Up and Go; UPDRS, Unified Parkinson's Disease Rating Scale; y, year.

^a^ One‐way ANOVA.

^b^ Chi‐square test.

^c^ Kruskal‐Wallis.

^d^ Two‐sample *t*‐test.

^e^ Mann–Whitney U.

* *p* < 0.001.

### 3.2 The Differences of Gait Parameters

A detailed description of the gait parameters for the different groups is summarized in Table 2.

**TABLE 2:** Results of two‐way ANOVA for gait parameters.

Gait parameters		MSA‐P (mean ± SD)	PD (mean ± SD)	HC (mean ± SD)	Group (MSA‐P vs. PD vs. HC)	Condition (STW vs. DTW)	Interaction (group × condition)
Step length (cm)	STW	47.75 ± 5.61	54.34 ± 6.54	55.82 ± 6.05	*F* = 21.461 ***p* < 0.001** *η* ^2^ = 0.254	*F* = 7.541 ***p* = 0.007** *η* ^2^ = 0.056	*F* = 0.073 p = 0.929 *η* ^2^ = 0.001
	DTW	44.69 ± 5.82	50.75 ± 8.68	53.29 ± 5.39			
Stride velocity (m/s)	STW	0.90 ± 0.09	0.90 ± 0.08	0.94 ± 0.13	*F* = 1.755 *p* = 0.177 *η* ^2^ = 0.027	*F* = 6.371 ***p* = 0.013** *η* ^2^ = 0.048	*F* = 0.093 *p* = 0.911 *η* ^2^ = 0.001
	DTW	0.86 ± 0.10	0.85 ± 0.08	0.89 ± 0.13			
Cadence (steps/min)	STW	107.49 ± 5.29	106.72 ± 6.92	105.90 ± 5.69	*F* = 2.080 *p* = 0.129 *η* ^2^ = 0.032	*F* = 3.838 *p* = 0.052 *η* ^2^ = 0.030	*F* = 0.349 *p* = 0.706 *η* ^2^ = 0.006
	DTW	110.74 ± 5.23	108.83 ± 6.10	107.35 ± 5.82			
Double support (%)	STW	19.43 ± 3.49	18.39 ± 2.21	18.43 ± 3.69	*F* = 0.882 *p* = 0.416 *η* ^2^ = 0.014	*F* = 2.137 *p* = 0.146 *η* ^2^ = 0.017	*F* = 0.121 *p* = 0.886 *η* ^2^ = 0.002
	DTW	19.89 ± 3.41	19.51 ± 2.46	19.27 ± 3.29			
Turning average duration (s)	STW	1.84 ± 0.50	1.81 ± 0.45	1.74 ± 0.50	*F* = 1.736 *p* = 0.180 *η* ^2^ = 0.027	*F* = 6.019 *p* = **0.016** *η* ^2^ = 0.046	*F* = 0.573 *p* = 0.565 *η* ^2^ = 0.009
	DTW	2.21 ± 0.61	1.98 ± 0.47	1.90 ± 0.65			
Turing average angular velocity (degree/s)	STW	104.59 ± 20.42	105.15 ± 20.42	110.25 ± 23.10	*F* = 2.355 *p* = 0.099 *η* ^2^ = 0.036	*F* = 6.461 *p* = **0.012** *η* ^2^ = 0.049	*F* = 0.540 *p* = 0.584 *η* ^2^ = 0.505
	DTW	88.92 ± 23.62	97.04 ± 22.99	103.79 ± 23.85			
Step length variability (%)	STW	8.78 ± 3.74	6.38 ± 1.70	4.99 ± 1.54	*F* = 11.571 ***p* < 0.001** *η* ^2^ = 0.155	*F* = 6.608 *p* = **0.011** *η* ^2^ = 0.050	*F* = 4.025 *p* = **0.020** *η* ^2^ = 0.060
	DTW	11.73 ± 4.34	6.60 ± 1.56	5.78 ± 1.70			
Stride velocity variability (%)	STW	7.77 ± 3.19	7.39 ± 2.93	6.37 ± 1.85	*F* = 0.958 *p* = 0.386 *η* ^2^ = 0.015	*F* = 14.806 ***p* < 0.001** *η* ^2^ = 0.105	*F* = 0.192 *p* = 0.826 *η* ^2^ = 0.003
	DTW	10.15 ± 5.23	9.70 ± 4.94	9.16 ± 3.64			
Cadence variability (%)	STW	7.83 ± 4.55	5.74 ± 1.45	5.94 ± 1.84	*F* = 8.892 *p* = **< 0.001** *η* ^2^ = 0.124	*F* = 1.480 *p* = 0.226 *η* ^2^ = 0.012	*F* = 0.271 *p* = 0.763 *η* ^2^ = 0.004
	DTW	8.98 ± 4.58	6.36 ± 2.03	6.14 ± 1.64			
Double support variability (%)	STW	0.18 ± 0.07	0.15 ± 0.08	0.15 ± 0.07	*F* = 1.616 *p* = 0.203 *η* ^2^ = 0.025	*F* = 2.251 *p* = 0.136 *η* ^2^ = 0.018	*F* = 0.276 *p* = 0.759 *η* ^2^ = 0.004
	DTW	0.20 ± 0.07	0.18 ± 0.08	0.16 ± 0.07			
Turning average duration variability (%)	STW	11.13 ± 0.11	9.19 ± 0.12	6.89 ± 0.08	*F* = 1.243 *p* = 0.292 *η* ^2^ = 0.019	*F* = 2.212 *p* = 0.139 *η* ^2^ = 0.017	*F* = 0.687 *p* = 0.505 *η* ^2^ = 0.011
	DTW	14.02 ± 0.13	9.21 ± 0.10	12.54 ± 0.12			
Turing average angular velocity variability (%)	STW	11.13 ± 0.11	9.19 ± 0.12	6.89 ± 0.08	*F* = 1.243 *p* = 0.292 *η* ^2^ = 0.019	*F* = 2.212 *p* = 0.139 *η* ^2^ = 0.017	*F* = 0.687 *p* = 0.505 *η* ^2^ = 0.011
	DTW	14.02 ± 0.13	9.21 ± 0.10	12.54 ± 0.12			

*Note:* Values are presented as the mean ± standard deviation. *p* < 0.05 was considered statistically significant. Values in bold are marked as significant.

Abbreviations: CV, coefficient of variation; DTW, dual‐task walking; HC, healthy control; MSA‐P, multiple system atrophy with predominant parkinsonism; PD, Parkinson's disease; STW, single‐task walking.

#### 3.2.1 Interaction Between Group and Condition

**FIGURE 1:**
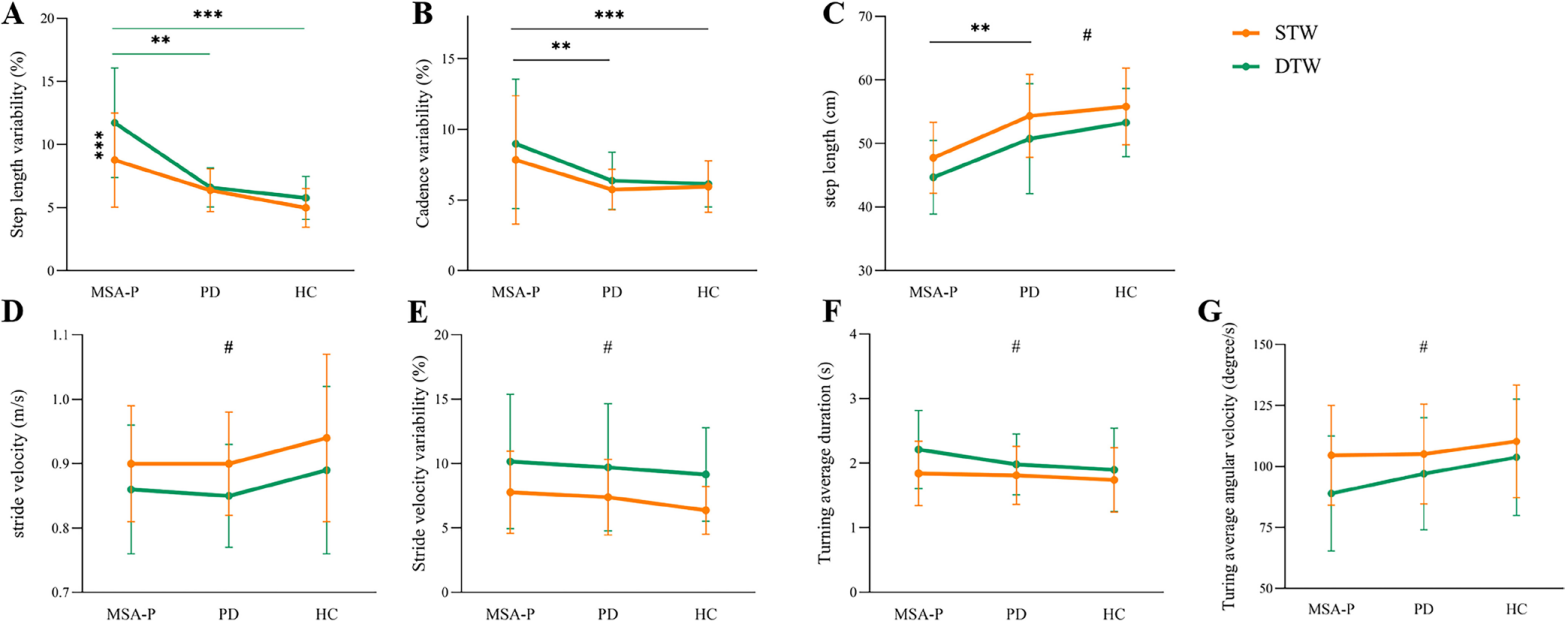
Effects of two walking conditions on gait parameters. (A) Interaction effect on the step length variability. (B, C) Main effect of group (MSA‐P vs. PD vs. HC) on the cadence variability and step length. (C–G) Main effect of conditions on the step length, stride velocity, stride velocity variability, turning average duration, and turning average angular velocity. A Bonferronicorrected threshold was set at *p* < 0.05. Details are shown in Table 2. **p < 0.01, ***p < 0.001, #A main effect of condition. DTW, dual‐task walking; HC, healthy control; MSA‐P, multiple system atrophy with predominant parkinsonism; PD, Parkinson's disease; s, seconds; STW, single‐task walking.

A significant interaction effect of group and condition was observed in step length variability, as indicated by the two‐way ANOVA (*F* = 4.025, *p* = 0.020, *η* ^2^ = 0.060, Figure 1A). Subsequent Bonferroni post hoc tests indicated that MSA‐P patients exhibited significantly higher step length variability compared to both PD patients (*p* = 0.001) and HCs (*p* < 0.001) in the DTW condition. Furthermore, MSA‐P patients demonstrated increased step length variability during the DTW condition compared to the STW condition (*p* < 0.001).

#### 3.2.2 Main Effect of Group

Significant main effects of the group were observed in both step length (*F* = 21.461, *p* < 0.001, *η* ^2^ = 0.254, Figure 1C) and cadence variability (*F* = 8.892, *p* < 0.001, *η* ^2^ = 0.124, Figure 1B). Further analysis using Bonferroni post hoc tests demonstrated that MSA‐P patients exhibited decreased step length (*p* < 0.001) and increased cadence variability (*p* = 0.002) compared to PD patients. Additionally, MSA‐P patients also displayed decreased step length (*p* < 0.001) and increased cadence variability (*p* < 0.001) when compared to the HCs.

#### 3.2.3 Main Effect of Condition

Significant main effects of the condition were observed for various gait parameters, including step length (*F* = 7.541, *p* = 0.007, *η* ^2^ = 0.056, Figure 1C), stride velocity (*F* = 6.371, *p* = 0.013, *η* ^2^ = 0.048, Figure 1D), stride velocity variability (*F* = 14.806, *p* < 0.001, *η* ^2^ = 0.105, Figure 1E), turning average duration (*F* = 6.019, *p* = 0.016, *η* ^2^ = 0.046, Figure 1F), and turning average angular velocity (*F* = 6.461, *p* = 0.012, *η* ^2^ = 0.049, Figure 1G). Bonferroni post hoc tests indicated that all participants exhibited increased stride velocity variability (*p* < 0.001) and turning average duration (*p* = 0.016) in the DTW condition compared to the STW condition. Furthermore, a decrease in step length (*p* = 0.007), stride velocity (*p* = 0.013) and turning average angular velocity (*p* = 0.012) was observed in all subjects during the DTW condition compared to the STW condition.

### 3.3 The Differences of ΔHbO_2_

**FIGURE 2:**
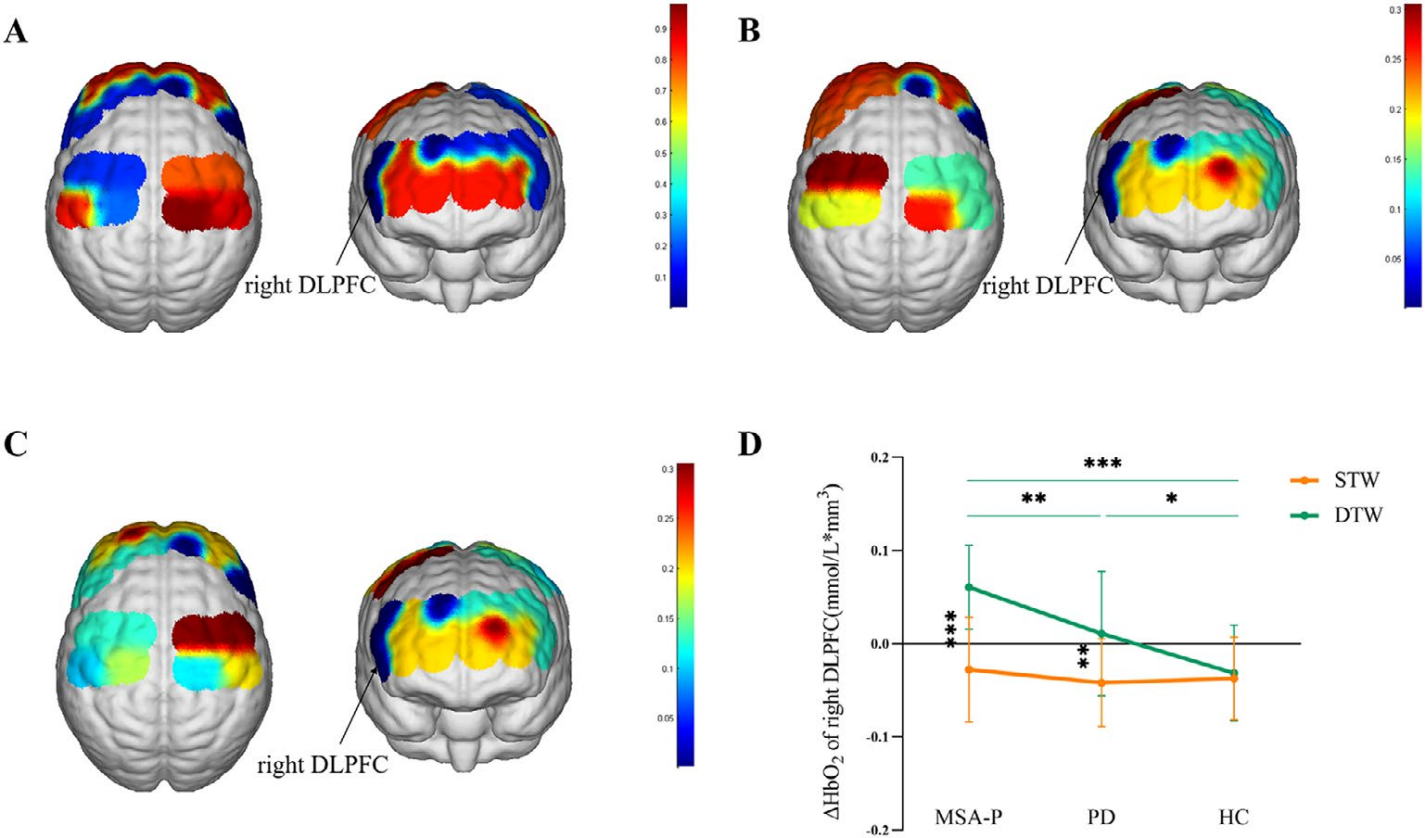
(A) Interaction between group and condition effect. The interaction between group and condition effect was found in the right DLPFC. (B) Main effect of group (MSA‐P vs. PD vs. HCs). Significant differences obtained from the main effect of group were in the right DLPFC. (C) Main effect of condition (STW vs. DTW). Significant differences obtained from the main effect of condition were in the right DLPFC. The color bar indicates *p*‐values from two‐way ANOVA, with group (MSA‐P vs. PD vs. HCs) and condition (STW vs. DTW) as the factors. The statistical threshold was set at *p* < 0.05 (Bonferronicorrected). (D) Post hoc tests in the right DLPFC. The ΔHbO_2_ in the right DLPFC in MSA‐P patients was significantly increased compared with that in PD patients (*p* = 0.006) and HCs (*p* < 0.001) in the DTW condition. PD patients showed increased ΔHbO_2_ compared to HCs (*p* = 0.028) in the right DLPFC during the DTW condition. In MSA‐P patients, an increased ΔHbO_2_ was observed in the DTW condition relative to that in the STW condition (*p* < 0.001). PD patients also exhibited a significant increase in ΔHbO_2_ in the DTW condition compared to the STW condition (*p* = 0.002). A Bonferroni‐corrected threshold was set at *p* < 0.05 for multiple comparisons. *p < 0.05, **p < 0.01, ***p < 0.001. ANOVA, analyses of variance; DLPFC, dorsolateral prefrontal cortex; DTW, dual‐task walking; HC, healthy control; MSA‐P, multiple system atrophy with predominant parkinsonism; PD, Parkinson's disease; STW, single‐task walking; ΔHbO_2_, the relative change in oxyhemoglobin during walking compared to the baseline.

A significant interaction effect between group and condition was identified in the right DLPFC (*F* = 7.196, *p* = 0.009, *η* ^2^ = 0.103, Figure 2A). Moreover, we also found a significant main effect of group in the right DLPFC (*F* = 11.193, *p* < 0.001, *η* ^2^ = 0.151, Figure 2B) and a main effect of condition in the right DLPFC (*F* = 28.720, *p* < 0.001, *η* ^2^ = 0.186, Figure 2C). For the interaction effect, Bonferroni post hoc tests (Figure 2D) indicated that MSA‐P patients exhibited a significant increase in ΔHbO_2_ compared to PD patients (*p* = 0.006) and HCs (*p* < 0.001) in the right DLPFC during the DTW condition. PD patients showed increased ΔHbO_2_ compared to HCs (*p* = 0.028) in the right DLPFC during the DTW condition. Additionally, within the MSA‐P group, an increased ΔHbO_2_ was observed in the DTW condition compared to the STW condition (*p* < 0.001, Figure 2D). Furthermore, PD patients also exhibited a significant increase in ΔHbO_2_ in the DTW condition compared to the STW condition (*p* = 0.002, Figure 2D).

### 3.4 Correlation of Gait Parameters With ΔHbO_2_

**FIGURE 3:**
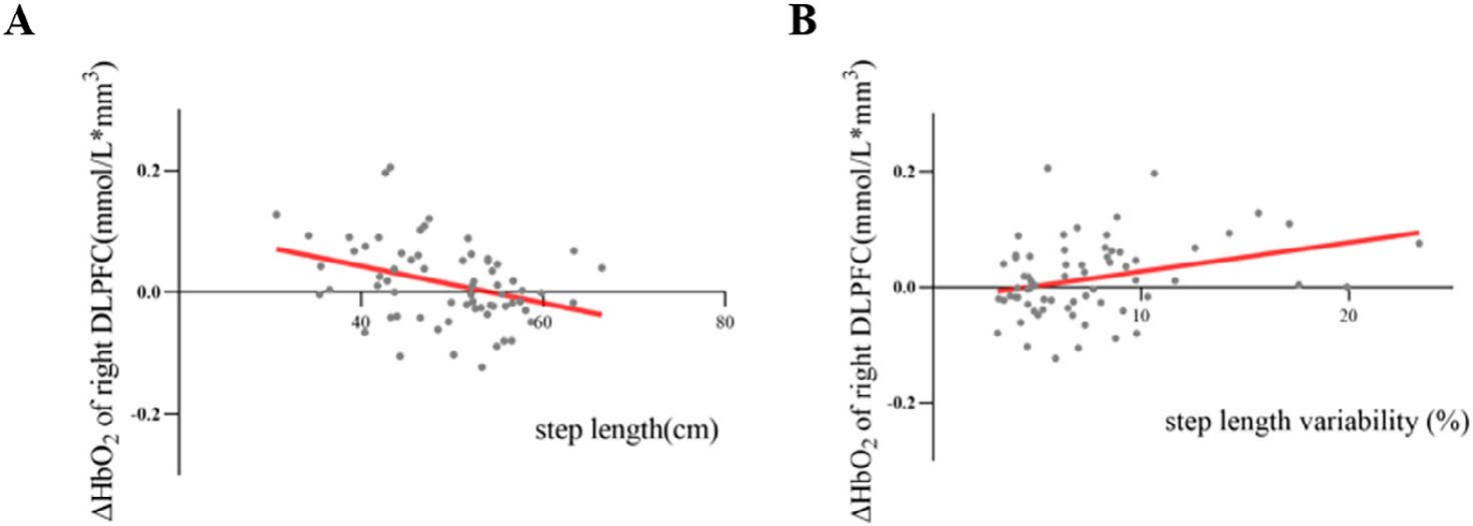
Correlations Between the ΔHbO_2_ in right DLPFC and gait parameters during DTW in All Participants. (A) Mild correlations between the ΔHbO_2_ in the right DLPFC and step length (*r* = −0.355, *p* = 0.003) during DTW. (B) Mild correlations between the ΔHbO_2_ in the right DLPFC and step length variability (*r* = 0.316, *p* = 0.010) during DTW. DLPFC, dorsolateral prefrontal cortex; DTW, dual‐task walking; ΔHbO_2_, the relative change in oxyhemoglobin during walking compared to the baseline.

Mild negative correlations were observed between the ΔHbO_2_ in the right DLPFC and step length (*r* = −0.355, *p* = 0.003) during DTW (Figure 3A). Moreover, we found mild positive correlations between the ΔHbO_2_ in the right DLPFC and step length variability (*r* = 0.316, *p* = 0.010) during DTW (Figure 3B). No significant correlations were found between the remaining gait parameters and ΔHbO_2_ in the right DLPFC during STW and DTW (Table S2).

### 3.5 ROC Curve Analysis

**FIGURE 4:**
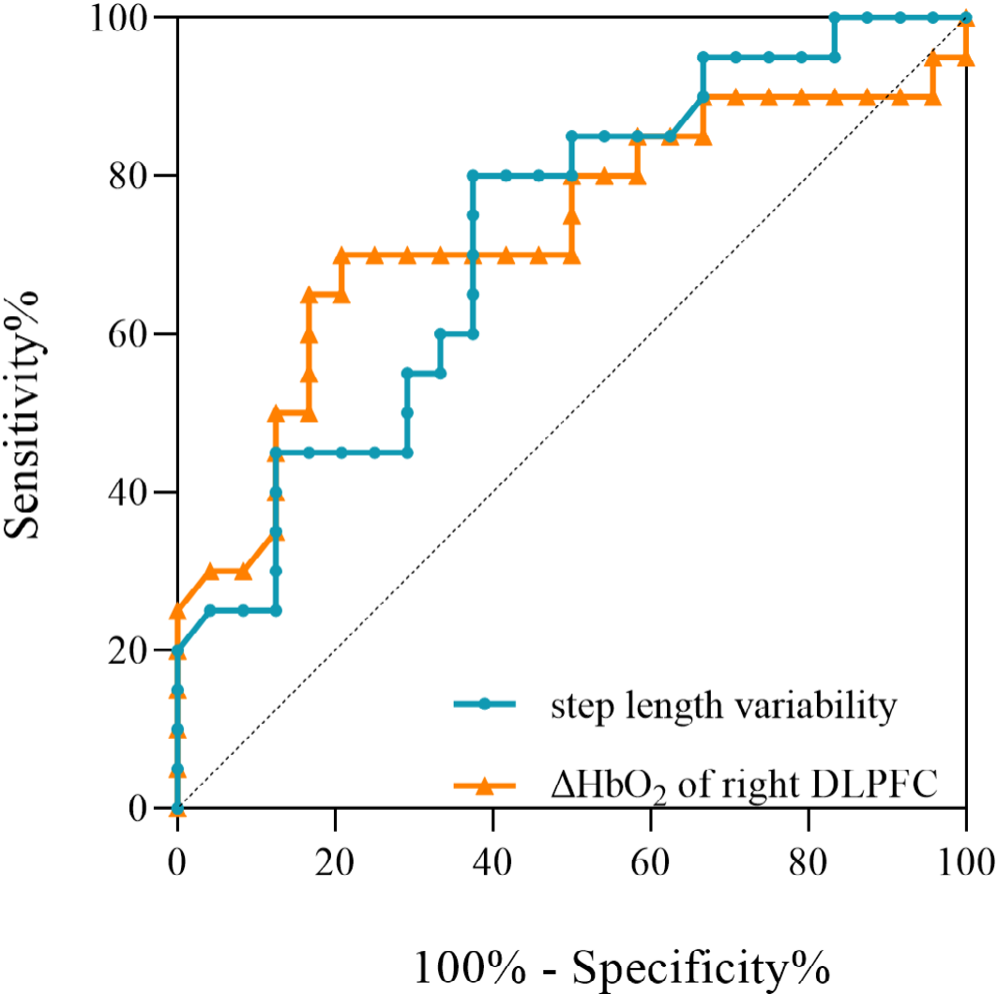
ROC analyses for differentiating MSA‐P from PD patients. The graph showed that the ΔHbO_2_ in the right DLPFC during DTW shows significant potential as an indicator for distinguishing MSA‐P and PD patients (AUC = 0.798, *p* < 0.001, 95% CI = 0.651–0.945, sensitivity = 0.650, specificity = 0.958, cut off point = 0.003). The step length variability during DTW also shows significant potential as an indicator for separating MSA‐P from PD patients (AUC = 0.721, *p* = 0.013, 95% CI = 0.570–0.871, sensitivity = 0.625, specificity = 0.800, cut off point = 8.215). See Table S2 for more detail. AUC, area under the curve; DLPFC, dorsolateral prefrontal cortex; DTW, dual‐task walking; MSA‐P, multiple system atrophy with predominant parkinsonism; PD, Parkinson's disease; ROC, receiver operating characteristic; ΔHbO_2_, the relative change in oxyhemoglobin during walking compared to the baseline.

ROC curve analyses showed that the AUC of step length variability during DTW was 0.721 when distinguishing the MSA‐P from the PD group (95% confidence interval [CI]: 0.570–0.871, *p* = 0. 013) (Figure 4 and Table S3). Meanwhile, the AUC of the ΔHbO_2_ in the right DLPFC during DTW was 0.798 (95% CI: 0.651–0.945, *p* < 0.001) (Figure 4 and Table S3).

## 4 Discussion

To the best of our knowledge, this study represents the first investigation into the cortical activity and gait impairment in individuals with MSA‐P as they perform cognitive tasks during walking and compares these findings with those of PD patients and a healthy control group. Firstly, our results indicate that MSA‐P patients exhibit higher step length variability and cortical activation during DTW compared to PD patients and HCs. Furthermore, we found mild negative correlations between the ΔHbO_2_ in the right DLPFC and step length during DTW, while there was a mild positive correlation between ΔHbO_2_ and step length variability during DTW. Additionally, ROC curve analyses uncovered that the ΔHbO_2_ of the right DLPFC and step length variability during DTW could be a potential features discerning MSA‐P patients and PD patients, respectively. Overall, our findings suggest that the gait disturbances experienced by MSA‐P patients are more severe than those of PD patients, potentially attributable to changes in the DLPFC.

Our study demonstrated that patients with MSA‐P exhibited increased gait variability and walked with greater irregularity during DTW, potentially leading to walking instability and an elevated risk of falling. According to current evidence, the decline in gait performance observed during DTW suggests that these tasks share limited cognitive resources [33]. In other words, engaging in these tasks concurrently results in competition for cognitive resources, ultimately leading to poorer performance in one or both tasks [34]. Consistent with prior research, our findings revealed that all three groups experienced impaired gait during DTW, with MSA‐P patients exhibiting particularly pronounced impairments. Therefore, we posit that MSA‐P patients may present underlying cognitive impairments, and the available cognitive resources may be insufficient to maintain optimal gait performance during complex walking. Consequently, this could contribute to the heightened gait impairment experienced by MSA‐P patients under conditions involving dual‐task walking.

Previous research suggests that the PFC, SMA, M1, and sensorimotor cortex are brain regions involved in gait performance. The PFC, focusing on the DLPFC, plays a crucial role in attention allocation and coordination [35, 36]. Zhang et al. [18] observed that individuals with PD showed higher relative changes in oxyhemoglobin in the left DLPFC compared to HC participants, and they exhibited unstable gait with a limited range of motion during walking. Moreover, multimodal imaging studies have indicated deficits in attention, executive function, and working memory in patients with MSA‐P, which were associated with reduced [^18^F]‐fluorodeoxyglucose ([^18^F]‐FDG) uptake in the DLPFC [37]. Consistent with these findings, our study demonstrated that MSA‐P patients had higher levels of ΔHbO_2_ in the right DLPFC and experienced more severe gait disturbances compared to PD patients during dual‐task walking. This suggests that MSA patients may rely on increased cortical activity to compensate for motor and cognitive deficits during complex walking tasks. Moreover, our study observed mild negative correlations between the ΔHbO_2_ in the right DLPFC and step length during DTW, while there was a mild positive correlation between ΔHbO_2_ and step length variability during DTW. Previous studies have reported similar findings examining usual walking [9]. These studies proposed an interpretation that higher activation in the PFC reflects inefficient motor adaptation. In other words, increased activation may represent a compensatory attempt to overcome inefficient neural activation in the face of demanding tasks.

In contrast, HC participants did not show significant changes in DLPFC activity between single and dual walking tasks, consistent with previous studies [9, 38]. Notably, PD patients and HC participants exhibited impaired gait performance during dual‐task walking. This could be attributed to prioritizing the subtraction task at the expense of the walking task or having sufficient cognitive resources to perform both tasks simultaneously. Furthermore, it has recently been suggested that patients with PD may rely on enhanced cognitive control, particularly executive control, to compensate for gait deficits [39]. Specifically, patients with more severe gait disturbances may require additional cognitive resources to manage motor symptoms. Based on the observed relationship between gait performance and cortical activity, we hypothesize that patients with mild motor symptoms require fewer cognitive resources for complex walking tasks or possess more effective compensatory prefrontal executive control over gait. Additionally, ROC curve analyses indicated that ΔHbO_2_ in the right DLPFC and step length variability during DTW could differentiate between MSA‐P and PD patients, suggesting their potential as clinical markers for distinguishing these two diseases.

One potential limitation of the present study is the constrained opportunity to recruit a larger number of participants due to the study being conducted in a single center. Additionally, it should be noted that fNIRS has good temporal resolution but limited spatial resolution compared to traditional neuroimaging methods like fMRI. Nonetheless, the use of portable fNIRS allowed us to capture functional alterations in the subjects' cerebral cortex during walking, which was highly beneficial for discerning differences among the three groups. Moreover, our fNIRS examination was performed after dopaminergic drug intake, which might modulate cortical function. However, LEDD was matched in the patients, which reduced the influence brought by the drug as much as possible. Furthermore, in our study, 5‐m was used as walking distance due to the spatial limitation of our lab. longer distance walking paradigms may be more representative, but walking distances as short as 15 ft have been found to produce reliable fNIRS data [38]. In future studies, it will be necessary to increase the sample size and refine the fNIRS analysis techniques.

## 5 Conclusions

This present study shows that MSA‐P patients demonstrated more severe gait disturbance and increased DLPFC activity compared with PD patients and HCs. This is possibly because increased DLPFC activation in MSA‐P patients is insufficient to compensate for motor and executive deficits. In conclusion, the study provided a new way to early identification of MSA‐P and PD patients.

## Author Contributions

Mengxi Gao: conceptualization, investigation, formal analysis, methodology, and writing the paper; Heng Zhang, Aidi Shan, and Yongsheng Yuan: investigation and validation; Xingyue Cao and Lina Wang: investigation and methodology; Caiting Gan, Huimin Sun, Shiyi Ye, and Chenghui Wan: investigation; Youyong Kong: conceptualization, supervision, and validation; Kezhong Zhang: conceptualization, funding acquisition, supervision, and validation.

## Conflicts of Interest

The authors declare no conflicts of interest.

## Supporting information


Data S1.

Data S2.

## Data Availability

The data that support the findings of this study are available from the corresponding author upon reasonable request.
