# Supplementary figures and images for: Differences Between Patients With Multiple System Atrophy With Predominant Parkinsonism and Parkinson's Disease Based on fNIRS and Gait Analysis
Source: CNS Neurosci Ther. 2025 Mar 26;31(3):e70342. doi: 10.1111/cns.70342 (PMC11937913; doi:10.1111/cns.70342)

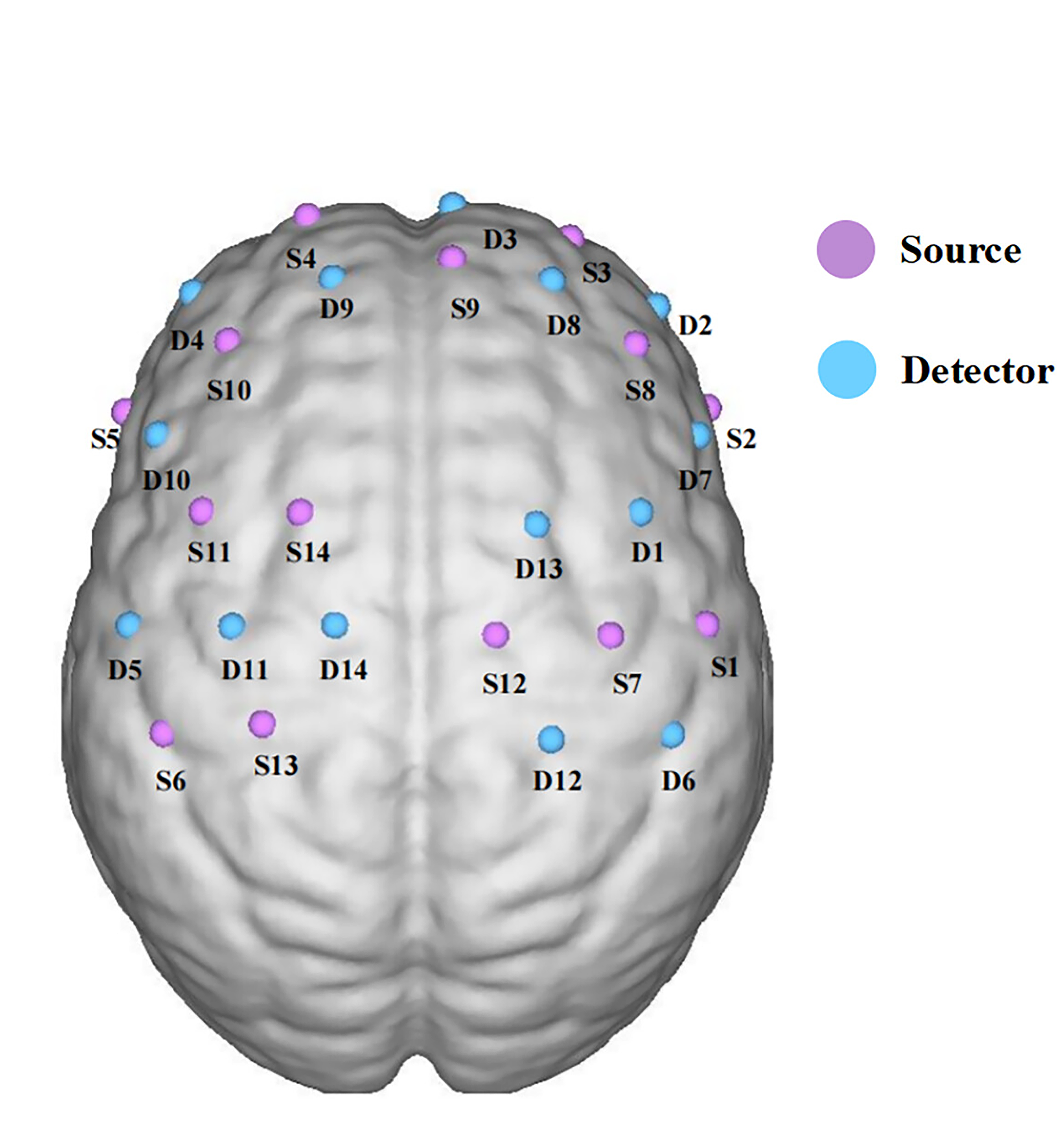

Supplement: Supplementary file 1 — Data S1. [file CNS-31-e70342-s001.tif]
