# Supplementary material for: Differences Between Patients With Multiple System Atrophy With Predominant Parkinsonism and Parkinson's Disease Based on fNIRS and Gait Analysis
Source: CNS Neurosci Ther. 2025 Mar 26;31(3):e70342. doi: 10.1111/cns.70342 (PMC11937913; doi:10.1111/cns.70342)
Supplement: Supplementary file 2 — Data S2. [file CNS-31-e70342-s002.docx]

**Supplementary materials**

**Table 1 The MNI coordinates and Brodmann areas corresponding to the measurement channels.**

| Channel | MNI coordinates | | | Brodmann areas (Talairach daemon) |
| --- | --- | --- | --- | --- |
|  | x | y | z |  |
| CH1 (S1-D1) | 52 | -2 | 55 | 6: Pre-Motor and Supplementary Motor Cortex |
| CH2 (S1-D6) | 56 | -24 | 57 | 2: Primary Somatosensory Cortex |
| CH3 (S2-D2) | 55 | 42 | 2 | 47: Dorsolateral Prefrontal Cortex |
| CH4 (S2-D7) | 60 | 29 | 14 | 45: Dorsolateral Prefrontal Cortex |
| CH5 (S3-D2) | 42 | 61 | 5 | 10: Frontopolar Area |
| CH6 (S3-D3) | 20 | 72 | 8 | 10: Frontopolar Area |
| CH7 (S3-D8) | 29 | 63 | 20 | 10: Frontopolar Area |
| CH8 (S4-D3) | -11 | 73 | 10 | 10: Frontopolar Area |
| CH9 (S4-D4) | -35 | 62 | 11 | 10: Frontopolar Area |
| CH10 (S4-D9) | -21 | 66 | 25 | 10: Frontopolar Area |
| CH11 (S5-D4) | -52 | 43 | 10 | 10: Dorsolateral Prefrontal Cortex |
| CH12 (S5-D10) | -56 | 29 | 24 | 10: Dorsolateral Prefrontal Cortex |
| CH13 (S6-D5) | -56 | -23 | 57 | 2: Primary Somatosensory Cortex |
| CH14 (S6-D11) | -45 | -23 | 67 | 3: Primary Somatosensory Cortex |
| CH15 (S7-D1) | 42 | -2 | 63 | 6: Pre-Motor and Supplementary Motor Cortex |
| CH16 (S7-D6) | 45 | -23 | 67 | 2: Primary Somatosensory Cortex |
| CH17 (S7-D12) | 34 | -24 | 73 | 4: Primary Motor Cortex |
| CH18 (S7-D13) | 31 | -5 | 69 | 6: Pre-Motor and Supplementary Motor Cortex |
| CH19 (S8-D2) | 47 | 49 | 18 | 10: Frontopolar Area |
| CH20 (S8-D7) | 52 | 36 | 29 | 46: Dorsolateral Prefrontal Cortex |
| CH21 (S8-D8) | 36 | 51 | 31 | 10: Frontopolar Area |
| CH22 (S9-D3) | 7 | 69 | 24 | 10: Frontopolar Area |
| CH23 (S9-D8) | 16 | 60 | 34 | 9: Dorsolateral Prefrontal Cortex |
| CH24 (S9-D9) | -8 | 61 | 37 | 9: Dorsolateral Prefrontal Cortex |
| CH25 (S10-D4) | -43 | 51 | 24 | 10: Frontopolar Area |
| CH26 (S10-D9) | -29 | 53 | 37 | 9: Dorsolateral Prefrontal Cortex |
| CH27 (S10-D10) | -46 | 35 | 38 | 9: Dorsolateral Prefrontal Cortex |
| CH28 (S11-D5) | -51 | -2 | 56 | 6: Pre-Motor and Supplementary Motor Cortex |
| CH29 (S11-D11) | -40 | -1 | 64 | 6: Pre-Motor and Supplementary Motor Cortex |
| CH30 (S12-D12) | 21 | -25 | 77 | 4: Primary Motor Cortex |
| CH31 (S12-D13) | 20 | -3 | 77 | 6: Pre-Motor and Supplementary Motor Cortex |
| CH32(S13-D11) | -35 | -22 | 74 | 4: Primary Motor Cortex |
| CH33 (S13-D14) | -23 | -21 | 77 | 4: Primary Motor Cortex |
| CH34(S14-D11) | -31 | -1 | 69 | 6: Pre-Motor and Supplementary Motor Cortex |
| CH35(S14-D14) | -19 | 0 | 76 | 6: Pre-Motor and Supplementary Motor Cortex |

Abbreviation: MNI, Montreal Neurological Institute; CH, Channel; S, Source; D, Decteor.

**Table 2 Correlation analysis between the ΔHbO_2_ in right DLPFC and gait parameters**

|  | ΔHbO_2_ in right DLPFC during STW | | ΔHbO_2_ in right DLPFC during DTW | |
| --- | --- | --- | --- | --- |
|  | r | p value | r | p value |
| Step length | -0.048 | 0.701 | -0.355 | 0.003***** |
| Stride velocity | 0.234 | 0.056 | 0.01 | 0.936 |
| Cadence | 0.089 | 0.482 | -0.001 | 0.996 |
| Double support | -0.003 | 0.984 | 0.098 | 0.429 |
| Turning average duration | -0.133 | 0.287 | 0.239 | 0.053 |
| Turing average angular velocity | 0.136 | 0.276 | -0.249 | 0.055 |
| Step length variability | 0.011 | 0.933 | 0.316 | **0.010*** |
| Stride velocity variability | -0.044 | 0.722 | -0.196 | 0.116 |
| Cadence variability | -0.126 | 0.316 | 0.163 | 0.190 |
| Double support variability | -0.034 | 0.789 | 0.025 | 0.844 |
| Turning average duration variability | -0.126 | 0.313 | 0.045 | 0.722 |
| Turing average angular velocity variability | -0.126 | 0.313 | 0.045 | 0.722 |

Note: Bold values are marked as significant, *p＜0.05 was considered statistically significant.

Abbreviation: STW, single-task walking; DTW, dual-task walking. DLPFC, dorsolateral prefrontal cortex;ΔHbO_2_, the relative change in oxyhemoglobin during walking compared to the baseline.

**Table 3 ROC analyses for differentiating MSA-P and PD patients.**

|  | AUC | *p* value | 95% CI | Sensitivity | Speciﬁcity | Cut-off point |
| --- | --- | --- | --- | --- | --- | --- |
| Step length variability in DTW | 0.721 | 0.013^*^ | 0.570-0.871 | 0.625 | 0.800 | 8.215 |
| ΔHbO_2_ in right DLPFC in DTW | 0.798 | < 0.001^*^ | 0.651-0.945 | 0.650 | 0.958 | 0.003 |

Note: Bold values are marked as significant, *p＜0.05 was considered statistically significant.

Abbreviation: ROC, receiver operating characteristic; AUC, area under the curve; CI, conﬁdence interval; DTW, dual-task walking; HbO_2_, oxyhemoglobin; PD, Parkinson’s disease; MSA-P, multiple system atrophy with predominant parkinsonism; DLPFC, dorsolateral prefrontal cortex;ΔHbO_2_, the relative change in oxyhemoglobin during walking compared to the baseline.

**Figure 1 The distribution of channels**

Note: Schematic illustration of the fNIRS layout (35 channels, 14 sources, and 14 detectors). The nodes represent optical probes. The arrangement covers the bilateral prefrontal cortex, pre-motor cortex, supplementary motor cortex, primary motor cortex, and primary somatosensory cortex. Details are shown in Table S1.

Abbreviation: fNIRS, functional near-infrared spectroscopy.
